# Challenges of Achieving Tuberculosis Elimination by 2050: A Need for More Attention in the TB Control Program in Iran

**Published:** 2017-05

**Authors:** Salman KHAZAEI, Shahab REZAEIAN

**Affiliations:** 1. Dept. of Epidemiology, School of Public Health, Hamadan University of Medical Sciences, Hamadan, Iran; 2. Research Center for Environmental Determinants of Health, Kermanshah University of Medical Sciences, Kermanshah, Iran

## Dear Editor-in-Chief

The epidemic of HIV and tuberculosis (TB) put a high socioeconomic burden on the communities. Despite progress in the TB program indexes in recent years, an increasing trend in HIV remains as a global challenge within and between countries. This letter aims to highlight a number of issues, which may help policymakers to make better policies to control the morbidity and mortality.

**Fig. 1:**
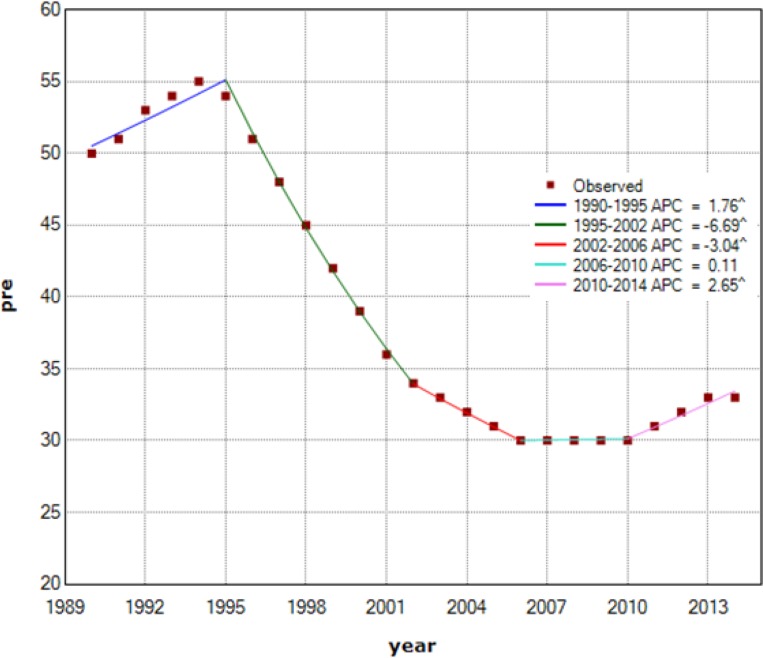
Trend and Annual Percent change (APC) for estimated prevalence of TB in Iran from 1990–2014

First, based on international reports of the WHO data for countries from 1990–2014 (Fig. 1), the estimated prevalence of TB from 1995 to 2010 in Iran was decreasing, after that there is an increasing trend for prevalence of TB (Annual Percent Change=2.65) (Table 1). In addition, the national studies have also showed an increasing trend in the TB incidence (1).

**Table 1:** Estimated prevalence of TB (all forms) per 100,000 populations in Iran during 1990–2014

**Year**	**1990**	**1995**	**2000**	**2005**	**2010**	**2014**
Prevalence	50 (25–82)	54 (27–89)	39 (20–64)	31 (16–51)	30 (15–50)	33 (17–55)

On the other hand, despite raised awareness about the HIV/AIDS in Iran some studies have shown an increasing trend in the HIV prevalence in the community and in the high-risk groups. A modeling study has shown an upward trend in the number of HIV infections in general population (2). Another study to determine the trend of HIV/AIDS prevalence in prisons during a 13-year period showed an increasing trend in the HIV infection during 2002–5 in Iranian prisons, but with a downward trend totally (3). Nevertheless, the prevalence of HIV was significantly higher in the prisons (3) where the high prevalence of TB has been reported. In other word, an 8-year study revealed an increasing trend in the TB incidence in Iranian prisons (4).

Another important issue, as a complicated factor to control TB, is related to the neighborhood countries of Iran. Through the periods of Iraq and Afghanistan war and now, Iran was a host country for the refugees who were susceptible to the disease. Accordingly, the increasing immigration trends could influence the prevalence and transmission patterns of TB in the host country (5).

As a result, the mentioned issues, which preclude any possibility of achieving TB elimination by 2050 need to be considered as, key factors affecting on TB prevention program.
